# Immunopathogenesis of patients with COVID-19: from the perspective of immune system ‘evolution’ and ‘revolution’

**DOI:** 10.1017/erm.2022.12

**Published:** 2022-04-12

**Authors:** Dan Lv, Bai Hu, Xingguang Lin, Renjie Wang, Di Wu, Rui Long, Mengzhou He, ShuJie Liao, Dongrui Deng

**Affiliations:** Department of Obstetrics and Gynecology, Tongji Hospital, Tongji Medical College, Huazhong University of Science and Technology, Wuhan, Hubei 430030, PR China

**Keywords:** Adults, autoimmunity, cancer, COVID-19, immunopathogenesis, SARS-CoV-2, sex, the aged, the young

## Abstract

The pandemic caused by severe acute respiratory syndrome coronavirus 2 is sweeping the world, threatening millions of lives and drastically altering our ways of living. According to current studies, failure to either activate or eliminate inflammatory responses timely and properly at certain stages could result in the progression of the disease. In other words, robust immune responses to coronavirus disease 2019 (COVID-19) are critical. However, they do not theoretically present in some special groups of people, including the young, the aged, patients with autoimmunity or cancer. Differences also do occur between men and women. Our immune system evolves to ensure delicate coordination at different stages of life. The innate immune cells mainly consisted of myeloid lineage cells, including neutrophils, basophils, eosinophils, dendritic cells and mast cells; they possess phagocytic capacity to different degrees at different stages of life. They are firstly recruited upon infection and may activate the adaptive immunity when needed. The adaptive immune cells, on the other way, are comprised mainly of lymphoid lineages. As one grows up, the adaptive immunity matures and expands its memory repertoire, accompanied by an adjustment in quantity and quality. In this review, we would summarise and analyse the immunological characteristics of these groups from the perspective of the immune system ‘evolution’ as well as ‘revolution’ that has been studied and speculated so far, which would aid the comprehensive understanding of COVID-19 and personalised-treatment strategy.

## Introduction

Severe acute respiratory syndrome coronavirus 2, briefed SARS-CoV-2 (Ref. 1), is the virus that has led to coronavirus disease 2019 (COVID-19), which threatens millions of lives worldwide. According to clinical manifestations, there are four main types of COVID-19 patients: asymptomatic, mild, severe and critical (Ref. 2). Critical cases often end in acute respiratory distress syndrome, Multiple organ dysfunction syndrome and even death. Severe and critical cases with SARS-CoV-2 are mainly characterised as older age, male gender, black patients and south Asians, smoking status and comorbidities including arterial hypertension, diabetes, obesity and chronic kidney disease, all of which are featured as chronic inflammation infiltration (Ref. 3). Besides, single-cell sequencing analysis of both peripheral blood (Ref. 4) and bronchoalveolar lavage fluid (Ref. 5) obtained from hospitalised patients with critical COVID-19 revealed a reduction in crosstalk between immune cells, but an increase in interplay between immune cells and structure cells. It indicates that aberrant resolution of inflammatory responses, also called ‘cytokine storm’ in many studies, contributes to disease progression and underlies the mechanism of tissue damage. The origin site of late-phase cytokine storm may not be the peripheral leucocytes, as there was a downregulation of proinflammatory cytokine genes accompanied by upregulation in exhaustion markers in immune cells (Ref. 4). To recap, we have concluded from published papers that patients with COVID-19 grouped by age and special immune characteristics have disparate outcomes. Therefore, it is vital to understand the immune characteristics at different stages of life.

## Brief introduction to the immunopathogenesis of SARS-CoV-2 infection

The immune system relies upon the cells of innate and adaptive immunity. Innate immune cells, which are not antigen-specific and lack immunological memory, include neutrophils, eosinophils, mast cells, basophils, monocytes, macrophages, natural killer (NK) cells and various types of dendritic cells (DCs). In contrast, the highly antigen-specific adaptive immunity, which characteristically develops immunological memory, is mediated by lymphocytes. These lymphocytes, which recombine antigen-receptor genes to generate diverse antigen recognition, comprise the helper, regulatory and cytotoxic T cells and the antibody-producing B cells.

After binding to the angiotensin-converting enzyme 2 (ACE2) receptor, SARS-CoV-2 gains its entry into target cells. Its spike (S) protein is primed by transmembrane protease serine 2 (Ref. 6). In the context of COVID-19, the main cells that SARS-CoV-2 invade in the lung tissue are alveolar epithelial cells, the other cells being neutrophils and macrophages, as unravelled by single-cell sequencing (Ref. 5). Membrane fusion happens, and virus RNA is released into target cells. Then virus RNA is translated utilising organelles of target cells. The infected cells send signals to pattern recognition receptors (PRRs) presenting on the antigen-presenting cells (APCs), leading to phagocytic activities by neutrophils, macrophages and the release of various pro-inflammatory cytokines and acute-phase proteins, such as C-reactio protein, interleukin 6 (IL-6), ferritin, by activated innate immune cells. Though PRRs are highly effective at discriminating self from non-self, they lack the specificity required to eliminate viruses that have managed to survive the initial defense (Ref. 7). Therefore, some viral proteins were simultaneously up-taken by APCs, mainly DCs, which professionally present processed peptides to T and B cells. The adaptive immune system amplifies and reinforces the innate immune response by providing cytokines, antibodies and cytotoxic T cells capable of dealing with virally infected cells. One of the profound signatures that adaptive immunity has is that it utilises genetically recombined receptors generated de novo to recognise infectious agents with high specificity and keeps the memory. The stockpile of such memory can be deployed fast upon a second attack, which is the reason for devoting tremendous efforts to developing vaccines to achieve herd immunity (Ref. 8). So far, a report by To *et al*. (Ref. 9) has showcased the re-infection of SARS-CoV-2. According to whole genome sequencing, the second episode of infection originated from another strain instead of prolonged viral shedding. Whether this indicates a short duration of natural immunity is still under discussion.

Collectively, haematological and biochemical parameters comparing differences between severe or not cases of disparate time points partially delineate the immune status of patients with COVID-19 and guide therapeutics. Compared with mild cases (Refs 10, 11), neutrophil–lymphocyte ratio (NLR) value increases significantly, and percentages of monocytes decrease as COVID-19 progresses; moreover, helper T (Th) cells and memory Th cells are lower in the severe group compared with that of mild groups, the opposite being observed as to naïve Th cell levels between these two groups. NLR is an easily accessed parameter from routine complete blood count. NLR elevation heralds inflammatory and infection status; it has been extensively studied in recent years and proven effective at providing the prognostic value in solid tumours (Ref. 12), sepsis (Ref. 13), autoimmune diseases (Ref. 14), metabolic syndrome (Ref. 15) and COVID-19 (Refs 16–18). Upon infection, there is a chemotactic influx of neutrophils to the lung; however, persistently high levels of neutrophil infiltration, which means the impromptu resolution of the virus, might cause collateral damage to surrounding tissue as seen in many chronic diseases. The accompanied lymphopaenia as a result of lymphocyte apoptosis ensuing critical immune dysfunction (Ref. 19), together with elevated neutrophil levels, indicates disease progression. However, NLR value can be influenced by many factors, including glucocorticoid use, smoking, obesity and alcohol use (Ref. 20). Future studies should incorporate stratification of confounding factors at a multicentre level before routine clinical use of NLR as a diagnostic and prognostic marker. Liao *et al*. (Ref. 21) showed that patients with severe-to-critical disease severity are more abundant in macrophages, neutrophils and lack of mDCs, pDCs and T cells than those with moderate severity. Single-cell spatial analysis of immune cells of post-mortem lung tissue obtained from patients with COVID-19 reveals that the absolute neutrophil numbers decrease at the late stage of infection compared with significantly increased macrophage populations (Ref. 5). Macrophages, derived from monocytes, are tissue-resident cells; they are immediately activated upon SARS-CoV-2 infection to phagocytise-infected cells and release cytokines, mainly pro-inflammatory phenotype including tumour necrosis factor-*α* (TNF-*α*), interferon-*β* (IFN-*β*), C-X-C motif chemokine 10 and monocyte chemoattractant protein 1 (MCP-1) to augment inflammatory responses (Ref. 22). They are critical in modulating disease severity during virus infection. Single-cell sequencing of bronchoalveolar lavage fluid (Ref. 21) revealed a highly pro-inflammatory macrophage phenotype in the lung of severe cases. The late stage of COVID-19 (defined by whether death occurs after 30 days from symptom onset) infection is found to be driven by the pathogen-independent immunological response. At this time, intra-cell-type interaction of immune cells decreased and interaction of macrophages with surrounding tissue cells increased, which underlies the mechanism of alveolar wall damage and resultant thickening (Ref. 5). Since pertinent studies exhibit conflicting results, the relationship between the NK cell quantity and the prognosis of COVID-19 remains to be explored (Refs 10, 23). However, Jiang *et al*. (Ref. 24) found that though NK cells decrease in quantity, their ability to respond to SARS-CoV-2 increases. As one recovers, the count and immune status of NK cells are restored to some degree.

Over-activation of cytokines, also called cytokine storm (Ref. 25), is caused by the imbalance in immune system regulation; it is observed in cohorts of severe-to-critical patients with COVID-19 (Ref. 26). TNF-*α*, L-1*β*, IL-2, IL-6, IL-17, IL-8 and MCP-1, all pro-inflammatory cytokines, are overproduced in severe cases (Ref. 27); together with ferritin, IL-10, they are strong discriminators for severe-to critical cases (Ref. 11). Notably, IL-6, IL-1*β* and TNF-*α* are important pro-inflammatory cytokines secreted by innate immune cells, mainly macrophages, mast cells, endothelial cells and epithelial cells (Ref. 28). These cytokines are known to augment inflammatory responses. IL-6 is the most extensively discussed because of its substantiated positive correlation with mechanical ventilation or death (Ref. 29). In an observational cohort studying rising kinetics of IL-6, Santa Cruz and colleagues (Ref. 30) found that though there are peaks in both survivors and non-survivors, IL-6 levels are significantly lower and decreased rapidly. The cut-off value suggested for worse outcomes is 86.95 pg/ml. Also, there are already approved drugs targeting IL-6 or its receptors. Monoclonal antibodies against IL-6 receptor (IL-6R) are therapeutic regimens proscribed for treating rheumatoid arthritis. They were later approved to treat cytokine release syndrome in chimeric antigen receptor T-cell therapies (Ref. 31). There are already clinical trials that have proved efficacy for critical cases of COVID-19 (Refs 32–35). Immunotherapies did not constantly achieve ideal effects (Ref. 36), which implies that multiple levels of cascades are involved. Further studies regarding dynamic surveillance of disease markers and their correlation with disease progression are warranted to guide drug use and medical resource allocation.

## Evolution of the immune system: faced with COVID-19

The immune system evolves to ensure delicate coordination at different stages of life. Foetuses live in a relatively sterile environment and should remain tolerant to semi-allograft maternal antigens; therefore, their total immunity status skews towards regulatory response (Ref. 26). After birth, their immune system develops as well in order to prepare making distinguish between ‘self’ and ‘outsiders’. One of the most profound characteristics is the peak of thymus function once puberty hits; after that, functional thymic epithelial space shrinks drastically. The thymus turns into fatty tissue at senility, accompanied by compromised adaptive immunity (Ref. 37). The innate immune cells are mainly comprised of cells of the myeloid lineage and NK cells of the lymphoid lineage. The adaptive immune cells, on the other way, are comprised mainly of lymphoid lineages. The development of the innate immune system precedes that of adaptive immunity in the foetal period. Mature neutrophils are first detected in the foetal bone marrow at 14 gestational weeks (Ref. 38). At around the seventh gestation week, parts of progenitor T cells migrate to the thymus and undergo maturation (Ref. 39). However, they are defective in both quantity and quality. Foetuses depend much on maternal antibodies – immunoglobulin G (IgG) (Ref. 40). Shortly after birth, the immune system develops fast in the face of mass pathogens. However, the immune responses tend to skew towards an anti-inflammatory profile (Ref. 26). As one grows up, the adaptive immunity matures and expands its memory repertoire, accompanied by the adjustment in quantity and quality. Because the aged undergo physiological changes in anatomy and biological function, their immune responses towards newly encountered pathogens are weak and insufficient compared with the young. There is little evidence of a natural halt of the SARS-CoV-2 infection before at least 50% of the population has been immune. Thus, active measures including self-quarantine, masking, social distancing and contact tracing must be stringently implemented to lower the virus-related reproductive number. By far, the ending of the pandemic relies on the adequate population-level herd immunity, which can be partially achieved by vaccines with efficient public control measures according to modelling and extrapolation from different studies (Ref. 41). Vaccines are more efficient in reducing viral circulation and overall mortality rates than naturally acquired herd immunity. To date, there are mRNA vaccines, virus vector vaccines, adjuvanted protein vaccines and live-attenuated and inactivated virus vaccines available. Most have achieved phase II/III results with scant data on group or age-stratified analysis. The current estimate of effective immunity to COVID-19 relies on detecting antibody titres to SARS-CoV-2, which does not cover the full spectrum of protective immunity. It has been reported that robust memory T cell responses exist without detectable circulating antibodies specific for SARS-CoV-2 (Ref. 42). This adds to the complexity of computation and management of COVID-19. The vaccines are initially prioritised to highly exposed populations and those at great risk of severe morbidity. As we have stepped into the vaccination era, it is inevitable to reflect on the long-term influence of the pandemic. Recently, the new term ‘long COVID’ emerged via social media (Refs 43–47). It is used to describe long-lasting effects of the infection or one or more of the usual symptoms that significantly impacted daily functions (Ref. 48); even patients with initially mild symptoms experience these post-traumatic events. The relevant health problems include breathing difficulties, enduring tiredness, reduced muscle function, impaired ability to perform daily vital tasks and mental health problems such as stress disorder, anxiety and depression. As it becomes more and more discussed, it has changed from patient-led efforts to scientific research, which pointed out that 87.4% of patients with COVID-19 had at least one symptom (Ref. 49). Of note, home-isolated young patients with mild COVID-19 are at risk of long-lasting dysponea and cognitive symptoms (Ref. 50). It is thus suggested that comprehensive infection control and population-wide mass vaccination be implemented. As surging studies have published satisfying results of COVID-19 vaccines, more groups of people are being considered as the targeted population. In the next section, we will summarise posited immune reactions to COVID-19 according to current findings and discuss their access to COVID-19 vaccines where relevant.

## Immune characteristics in the perinatal period: immature but promising

The innate response dominates in the newborn, although their ability to make antibodies has partially developed by the time of labouring. Since the fifth gestation week, foetuses start to produce innate immune cells of myeloid lineages such as neutrophils (Ref. 51). Neonates exhibit a relatively high neutrophil count at birth because of antepartum provocation of granulocyte-colony-stimulating factor (Ref. 52). However, most of them are immature and are incapable of migrating, phagocytising and digesting pathogens; but the condition changes with the stabilisation of neutrophil counts 72 h after birth. Furthermore, lymph nodes and spleen remain relatively underdeveloped in humans at the time of birth. Monocytes accrue in the first 2 weeks of life and decline from the third postnatal week (Ref. 53). Their phagocytic activity is not compromised compared with adults (Ref. 54), but they preferentially express the M2 signature (Ref. 51). NK cells are effector lymphocytes of the innate immunity and can elicit either cytotoxic or apoptotic effects on targeted cells. It is achieved by properly monitoring major histocompatibility complex class I molecules expressed on host cells. NK cell count elevates within 5 days post-partum and drastically declines on the following days (Ref. 55). Similar to compromised neutrophil and monocyte functions in neonates, NK cells are relatively low cytotoxic for their decreased ability to degranulate (Ref. 56). Neonates show discrepant Toll-like receptor (TLR) responses and cytokine production compared with adults. In vitro studies revealed poor activation of type 1 T helper cells (Th1) and low level of pro-inflammatory cytokines such as IL-1*β* and TNF-*α* upon PRR stimulation (Ref. 51); this indicates inadequate responses in the face of intracellular pathogens. CD4^+^ T helper cells are considered to skew towards Th2 because of increased IL-4 and IL-10 secretion, both of which suppress IFN-*γ* production (Ref. 57). Compared with adult APCs, there is impaired production of IL-12p70 in those of neonates (Ref. 57), which testified impaired Th 1 polarisation in vivo.

Th1 is considered vital in antiviral and antibacterial responses; thus, it is accepted that protective mechanisms at the perinatal period are compromised. However, Gibbons *et al*. (Ref. 58) found that though constituents of immune cells in neonates are discrepant from that of adults, neonates can initiate effective responses towards pathogens through IL-8-secreting T cells, which are deficient in adults. Their B-cells are naïve, lack antigenic exposure and have only a partially developed surface immunoglobulin repertoire. They tend to become memory cells instead of antibody-producing plasma cells. Except for IgG, an antibody that can be transferred trans-placentally from mother to foetus, other immunoglobulin levels are relatively low in the absence of intrauterine infection. To recap, innate and adaptive immunity in neonates is still immature and unprepared for the bulk of new pathogens encountered, although part of their responses has been established in the uterus. Meanwhile, they have an extensive repertoire of naïve cells waiting to be trained. Pneumonia is the most common infectious disease in the newborns (Ref. 59). It is reported that the occurrence of pneumonia is related to the weakened immunity of newborns, such as low levels of IL-2 in neonates with pneumonia, impaired number of CD4^+^ T and CD8^+^ T lymphocytes, and decreased levels of IgM and IgG antibodies (Ref. 60).

Since there are proven intrauterine infections upon congenitally transmitted TORCH (acronym for Toxoplasma, other, rubella, cytomegalovirus, herpes) agents via the haematogenous route (Ref. 61), there are concerns that the foetuses may be infected with SARS-CoV-2 through intrauterine vertical transmission. Many studies have detected amniotic fluid, placenta tissue, vaginal swab, cord blood, postpartum nasopharyngeal swab, faeces, and the urine of the neonates to evaluate such conditions (Refs 62–64). The positive rate of throat swabs of neonates born to mothers with COVID-19 ranges from 4.2% (35/836) to 6.6% (3/45). SARS-CoV-2 IgM and IgG antibodies in cord blood can be detected in some neonates (Ref. 65); however, all the reverse transcription-polymerase chain reaction (RT-PCR) tests are negative (Ref. 63). One case reported the positive RT-PCR test of amniotic fluid, and the pregnant woman was diagnosed as severely infected (Ref. 66). The results above indicate that although intrauterine infection of SARS-CoV-2 is possible, the rate is low. Preterm birth rates are high in various retrospective studies. Because pregnancy with COVID-19 is prone to elective termination in late pregnancy, many confounding factors must be adjusted before considering that SARS-CoV-2 infection may significantly add to inflammation-driven preterm labour. Besides, there are many other detailed COVID-19-related causal effect relationships in observational studies to be confirmed. Accompanied by data on the perinatal outcomes of COVID-19 infection, there is surging research into deciphering the underlying mechanism. As was introduced, SARS-CoV-2 gains its entrance into targeted cells through binding to ACE2. Recently, bioinformatics analysis of single-cell RNA expression profile of ACE2 and TMPRSS2 in the human trophectoderm and placenta of first and second trimester pregnancy by Cui *et al*. (Ref. 67) disclosed that ACE2 and TMPRSS2 are expressed in the first (67.9 and 63% respectively) and second (30 and 20.1% respectively) trimesters. The expression profile of ACE2 and TMPRSS2 in the third trimester was also confirmed (Ref. 68). Functional analysis of ACE2 + TMPRSS2 positive cells in the first trimester trophectoderm cells shows a strong relationship with viral invasion capacity, epithelial cell proliferation and cell-adhesion-molecule binding; ACE2 + TMPRSS2 positive cells in the second trimester extra-villous trophoblast exhibit association with branching structure morphogenesis, extracellular matrix interaction, oxygen binding and antioxidant activity. The findings suggest a physiological basis of SARS-CoV-2 invasion at the maternal–foetal interface and that possible intrauterine infection of SARS-CoV-2 might result in placenta insufficiency that leads to pregnancy complications. Future research should be targeted to decipher the overarching trophoblastic immune responses at different trimesters of pregnancy. To recap, neonatal outcomes are favourable. Although perinatal deaths occur, there is no causal relationship with COVID-19 (Ref. 69). Neonates with positive RT-PCR results for SARS-CoV-2 were either asymptomatic or mild (Ref. 70). The trophoblastic immune responses after SARS-CoV-2 infection and its long-term effects on neonatal development are still unknown.

## Immune characteristics in childhood and puberty: developing and vibrant

Since adaptive immunity is underdeveloped, children still rely much on the innate immune system. NK cell number exhibits decremental changes until 5 years of age when it reaches the adult level (Ref. 55). Before adulthood, NK cells of children go through upregulation in killer cell immunoglobulin-like receptor and concomitant downregulation of NKG2A, an inhibitory receptor, which means the enhancement of cytotoxic activity. Teran *et al*. (Ref. 71) found decreased repertoire of naïve CD4^+^ T cells and increased memory CD4^+^ and CD8^+^ T cells in children as they grow until 5 years of age, suggesting activation of their adaptive immunity. Concomitant changes have been observed in the reduction of pro-inflammatory cytokine secretions upon TLR recognition and activation. As is introduced above, the immune system in children is still developing but is far intense than that in neonates.

Though being at the same risk of infection with SARS-CoV-2 as the general population (Ref. 72), most children and adolescents who were confirmed cases of SARS-CoV-2 present with mild symptoms, and their risk of disease severity is not affected by age and gender. Early observational studies suggested that children and adolescents get infections mainly from family clusters (Ref. 72) and are more likely to obey health instructions such as facial masks and social distancing by parents and health care providers. Recently, it has been believed that children may play a significant role in transmission because of their asymptomatic presentation and less frequent testing in most cases (Ref. 73). Similar to adults, children presented to hospitals with severe symptoms are prone to be obese and have a high pro-inflammatory profile in serum (Ref. 74). The underlying immunological mechanisms for the differences (milder symptoms, better outcomes in children and adolescents than in adults) may be the low level of pro-inflammatory factors and the low likelihood of over-triggering immune responses. Currently, there are no completed phase III vaccination trials targeted at children and adolescents. The main interests and considerations of vaccination in this group are safety, acceptance, unknown dose, immunogenicity and adverse outcomes (Ref. 75).

## Immune characteristics in sexual maturity: mature and differentiated

Males and females differ in the face of infection and autoimmunity, suggesting possible distinctions between their immune systems. Sex hormones are deemed major disparities between these two groups. It was confirmed in the mouse model of the Middle East respiratory syndrome-coronaviruses (Ref. 76), where male mice showcased more pronounced susceptibility and disease severity than female mice, and the differences were more significant as age increases. Indeed, it is noteworthy that both oestrogen receptors and androgen receptors are present on various immune cells such as neutrophils, macrophages, NK cells, DCs, B and T cells (Ref. 77). Although investigations into the role of oestrogen in immune responses have frequently led to contradictory data, it has been found to stimulate Th cells, B cells, NK cells and Treg (Ref. 78) cells in total. Also, physiological changes in the E2 level such as the menstrual cycle (Ref. 79), pregnancy (Ref. 78), menopause are often associated with undulant autoimmune diseases. Castration of post-pubertal male mice showcases an increase in T cell levels in secondary lymphoid tissues and enhanced T cell proliferation (Ref. 80). Bongen *et al*. (Ref. 81) found female-associated immune sex expression signature (iSEXS) genes were enriched for genes encoding CD4^+^ T-cell, which may be responsible for high proportions of CD4^+^ T cells in the blood; male-associated iSEXS genes were abundant in myeloid-cell-expressed genes, leading to age-dependent sex differences in high monocyte proportions. Changes in immune system characteristics between males and females directly or indirectly resulting from hormone levels may partially indicate that women are prone to exert anti-inflammatory effects on infection.

It is observed that men are at a higher risk of being severely infected than women. As ACE2 and TMPRSS2 are responsible for SARS-CoV-2 entry into cells, some gender-related studies try to find the association between genetic polymorphisms and expression differences of the two genes and gender. ACE2 gene is located on the X-chromosome; thus, women are putatively to have better clinical outcomes because of potential heterozygous phenotype (Ref. 82). ACE2 expression is higher in men than women because men are prone to have smoking habits, which significantly upregulates ACE2 expression in the lung tissue (Ref. 83). Besides, a previous study has substantiated that orchiectomised male mice have reduced cardiac ACE2 activity, and ovariectomised female mice have increased ACE2 activity (Ref. 84). Future studies regarding ACE2 expression in the lung and heart tissues are warranted. It is implied that sex hormones are vital in regulating ACE2 expression. The higher expression of TMPRSS2 in men may be explained by their higher androgen level (Ref. 85). Androgen-deprivation therapies partially protected them from SARS-CoV-2 infection (Ref. 86), but its correlation with TMPRSS2 expression has yet to be decided. A large observational study by Gudbjartsson and colleagues (Ref. 87) disclosed that women also had lower anti-spike and anti-nucleoprotein antibodies than men, and women were less sick. Humoral immune responses are critical in aiding the clearance of SARS-CoV-2. However, the current understanding of the capacity to elicit effective humoral immunity is primarily dependent on the antibody titres and commercial assays (mainly anti-spike and anti-nucleoprotein). It is not sure whether these two markers are associated with protective immunity. Also, the waning of antibody titres does not mean the dampening of plasma cells to produce antibodies (Ref. 88). Thus, the results are unadjusted by many confounding factors and should be interpreted with caution. The study by Takahashi and colleagues (Ref. 89) made comparisons of viral loads and blood cell phenotyping between men and women. They found that male patients had higher plasma levels of innate immune cytokines, and more robust induction of non-classical monocytes, whereas female patients had more robust T cell activation. Female patients with robust innate immune responses towards SARS-CoV-2 tend to have poor outcomes, not observed in male patients. Thus, sex-specific approaches to COVID-19 diagnosis, treatment and vaccination merits consideration.

## Immune characteristics in the aged: vulnerable

Ageing is associated with a general reduction of immune functions and paradoxically escalated pro-inflammatory cytokine levels such as TNF, IL-6 and IL-1*β* (Ref. 90). This may result from diminished ability to resolve the infection. Lymphocytes in the aged exhibit increased sensitivity to TNF-*α*-induced apoptosis (Ref. 91) via decreased activation of the nuclear factor-*κ*B pathway. It was reported that the aged exhibit lower proportions of classical CD14^+^CD16^−^ monocytes compared with the young but increased levels of mDC2 (Ref. 92). Since mDC2 populations respond to TLR-3, -7, -8 and -9, it is reasoned that this is compensation for diminishing viral surveillance. Their neutrophils have defects in migration, phagocytosis and capturing pathogens using neutrophil extracellular traps (Refs 93, 94). One of the most profound characteristics of immunosenescence in adaptive immunity is the reduction in peripheral naïve CD8^+^ T cell counts and elevation in proportions of memory CD8^+^ T cells which are productions after multiple times of division (Ref. 95). Continuous exposure to antigen in adulthood and thymus involution accelerate the drainage of naïve T cells. The thymus is the largest at birth than at other time points. It remains active until puberty (around 10) and then starts to involute. Thymic epithelial space falls over time to less than 20% of the total space by 5 years of age; fatty tissues then replace it as it ages (Ref. 37). Certain critical events in thymus-dependent T cell development are largely set perinatally, as neonatal subtotal thymectomy is not associated with autoimmunity (Ref. 96) or susceptibility to infection (Ref. 97) later in life. However, compared with individuals with intact thymus, those who experienced subtotal thymectomy have lower levels of naïve T cells (Ref. 98) replenished by the thymus and are vital in the face of new pathogens. Thymus involution occurs at puberty and ends when immunosenescence happens, and the thymus is not as productive as before. Taken together, the aged are more vulnerable to new pathogens than the young because of their extensively decreased repertoire of naïve cells. There is a preferential anti-inflammatory cytokine profile of IL-10 secretion that aids the decline in CD8^+^T (Ref. 99). In addition, reduction in naïve T cell quantity makes it difficult for CD8^+^T to undergo clonal proliferation. These naïve T cells decline in quantity and quality in the aged, and simultaneously CD8^+^ T possesses lower expression levels of CD28, a co-stimulatory molecule indispensable for activation, in the elderly than those in the young, which makes the former more vulnerable to viral infection. Compared with a significant reduction in CD8^+^ T cells, there is a lesser extent in the undulance of B cells and neutrophils (Ref. 100). IgM levels, together with IgG and IgA antibody levels, increase in the aged. The increase in total IgG and IgA antibody levels may reflect secretion by bone marrow plasma-blasts of antibodies with limited somatic hypermutation. However, their vaccine responses are diminishing and have a rapid waning of antibodies (Ref. 100).

As mentioned, in patients with COVID-19, age, male sex, ethnicity and comorbidities are risk factors for predisposition and progression, addressed in many cohorts (Refs 101, 102). Age is the most significant independent risk factor for severity and morbidity. Ageing is characterised by increased baseline pro-inflammatory levels because of over-production of sterile inflammation upon clearing senescent cells and diminished ability to resolve inflammation. The consequence is that amplified immune responses can be produced, which can be excess and devastating (Ref. 103). In addition, evidence is that there is a negative correlation between age and numbers of fully activated T cells (Ref. 89). Thus, the aged should be considered both baseline pro-inflammatory characteristics and COVID-19 as to treatment. They are not the primary target of the population to be vaccinated against COVID-19 because it is expected that vaccines may not be as efficacious or safe for the aged. The conundrum thus lies between the high risk of morbidity and mortality and sparse data regarding treatment and vaccination. The two vaccines that proved to be most efficacious are Pfizer/BioNTech (Ref. 104) and Moderna (Ref. 105), both mRNA vaccines. They have reached efficacy over 90% and been reported the phase III results. However, little data have been released on the aged patients. Yet, a phase III study incorporating adult patients aged 18–84 might yield results on serostatus and adverse effects of the aged after vaccination.

The immune system works upon recognition of non-self-antigens. However, there are dilemmas that antigens are neither promptly recognised nor adequately recognised. Our immune system ‘revolutionises’ in these unwanted statuses and does not function to elicit robust or proper responses. Conditions of patients with SARS-CoV-2 are complicated by cancer or autoimmunity, either of which causes drastic changes to the immune system. Next, we will discuss the immunopathogenesis of SARS-CoV-2 infection in these two unique cohorts.

## Immune alterations about COVID-19 in patients with autoimmune diseases

Given that autoimmune diseases often arise at middle age or later, it is speculated that both genetic predisposition and environmental exposure contribute to the pathogenesis of autoimmunity. In this condition, T and B cells are abnormally activated by self-antigens, which lead to mass production of downstream autoantibodies and pro-inflammatory cytokines. According to the distribution of autoantigen, autoimmunity can be classified into two categories: organ-specific and non-organ-specific. The former is known for Hashimoto's disease and primary biliary cirrhosis, where there is a specific lesion of lymphocytes infiltration; the latter, on the other hands, is representative of diseases such as rheumatoid arthritis, systemic lupus erythematosus (SLE) and ankylosing spondylitis, all of which are characterised by systemic inflammation. Tolerance between lymphocytes and self-antigen in autoimmunity is breached during this period. B cells are fundamental in immune responses because they effectively secret antigen-specific antibodies. Briefly, they are classified into CD5^−^ conventional B cells and CD5^+^B1 cells. CD5^+^B1 cells produce low-affinity IgM auto-antibodies that constitute our natural antibody spectrum and help with the clearance of ageing or damaged cells (Ref. 106). They decrease in ageing physiologically but are aberrantly abundant in patients with autoimmune diseases, such as rheumatoid arthritis, SLE and autoimmune thyroid diseases (Ref. 107).

There are similar parts between patients with COVID-19 and autoimmune diseases, including dysregulated immune responses, corticosteroid application and monoclonal antibodies against pro-inflammatory cytokines. Autoimmune diseases do not seem to increase the predisposition to COVID-19 (Ref. 108) but aggravate disease severity in hospitalised patients with COVID-19 (Ref. 25). Pascolini and colleagues (Ref. 109) detected the existence of at least one autoantibody in patients with COVID-19 (15/33, 45%) and found that the presence of autoantibody worsens the disease course of COVID-19. It is observed that patients with COVID-19 are prone to have autoimmune diseases such as cold agglutinin syndrome (Ref. 110), autoimmune haemolytic anaemia (Ref. 111), Guillain–Barré syndrome (Ref. 112) and SLE (Ref. 113). Thus, tissue damage as a result of abnormal inflammation, iatrogenic effects of immunosuppressive drugs and general susceptibility to SARS-CoV-2 together put a threat on weighing the balance between routine treatment and possible risks. The fact that COVID-19 may aggravate and trigger autoimmune diseases makes patients with preexisting immune dysfunction not prioritised to a vaccination before careful evaluation.

## Possible immune responses to SARS-CoV-2 in patients with cancer

The external environmental factors and resultant genetic mutations are responsible for carcinogenesis. The growing cancer cells redirected cellular growth mechanisms to deprive normal cells of nutritional resources for their utility. During this enduring process, tumour antigens that are strongly recognised by our immune system are usually wiped out, leaving ‘transformed’ antigens under-recognised by the immune system. Thus, robust immune responses and adaptive immunity education cannot be achieved. Moreover, inflammation worsens the condition by enhancing tumour growth and survival. Together, tumour cells deprive normal cells of nutrients and space that are indispensable for growth and escape the immune system's attack via several mechanisms, including immunoediting that leads to low immunogenicity and anergy of T cells through engaging immune checkpoint molecules such as CTLA-4 and PD-1 on T cells. Cancer immunotherapies are targeted on putative theories mentioned above, including passive immunity using monoclonal antibodies against molecules expressed highly on tumours, monoclonal antibodies together with toxins, monoclonal antibodies against PD-L1, PD-1, CTLA-4 and so on. To recap, the immunosuppressive status can be acquired because of both cancer and cancer therapies.

The interactive burden of SARS-CoV-2 infection and cancer on patients hence received attention. A meta-analysis conducted by Desai *et al*. (Ref. 114) disclosed that the overall mortality rate was 30% among hospitalised patients with both COVID-19 and cancer, higher than that of the general hospitalised population (Refs 115, 116), which ranges 21–22%. It is (Refs 114, 117) also disclosed that increasing age, male sex, haematologic malignancy (risk ratio: 2.68, 95% confidence interval: 1.90–3.78, *I*^2^ = 0%, *P* < 0.00001), and current cancer therapy contribute to high mortality rates among this cohort. These data, however, should be interpreted with caution because whether patients with COVID-19 and cancer are more likely to die compared with patients with sole COVID-19 are based on the time of diagnosis, cancer type and immunosuppressive status caused by treatment, which would be significant confounders to disease course and prognosis. To recap, cancer patients' severity- and treatment-stratified observational studies are still warranted. Safety and efficacy issues in cancer patients also hamper vaccine administration in this group.

## Concluding remarks

COVID-19 is a newly emerging pandemic that threatens millions of lives and has drastically altered people's ways of living since the end of 2019. Though most of its epidemiological traits have been uncovered and applied to general guide, long-term research is still warranted for population-stratified precise medication. In this review, we have summarised disparate outcomes after SARS-CoV-2 infection among people of different ages and diverse backgrounds. The intrinsic reasons may have originated from their unique immune backgrounds. Though details concerning immunopathogenesis of SARS-CoV-2 infection among patients at different stages of life remain to be fully explored, extrapolation can be made from existing knowledge. Diagnosed intrauterine infection is rare, and outcomes of neonates and children diagnosed COVID-19 are often favourable, which may be because of their underdeveloped anatomy and immature but promising immunological characteristics. Though both are immunocompetence, women are less likely to be affected than men. It is believed that oestrogen and androgen, the most apparent difference between them, play pivotal roles. Immune status in the aged is deemed compromised from immune senescence and increased baseline inflammation, which renders them vulnerable and intricate upon SARS-CoV-2 infection. From the perspective of evolution, the unique anatomy and physiology of the immune system at different ages might be protective to individuals; however, the variation also determines their discrepant responses to pathogens. It also raises challenges to health care providers that age-specific stratification of patients with COVID-19 is required. Moreover, patients with either autoimmunity or cancer are still full of heterogeneity. Thus, they are often ruled out from clinical trials, such as vaccination. There is still much to uncover as we step into the vaccination era. The high incidence and profound impact caused by ‘long COVID’ deserves more research for medical counselling and pain alleviation.
